# Supplementary figures and images for: Environmental variables influence patterns of mammal co-occurrence following introduced predator control
Source: PLoS One. 2023 Nov 30;18(11):e0292919. doi: 10.1371/journal.pone.0292919 (PMC10688647; doi:10.1371/journal.pone.0292919)

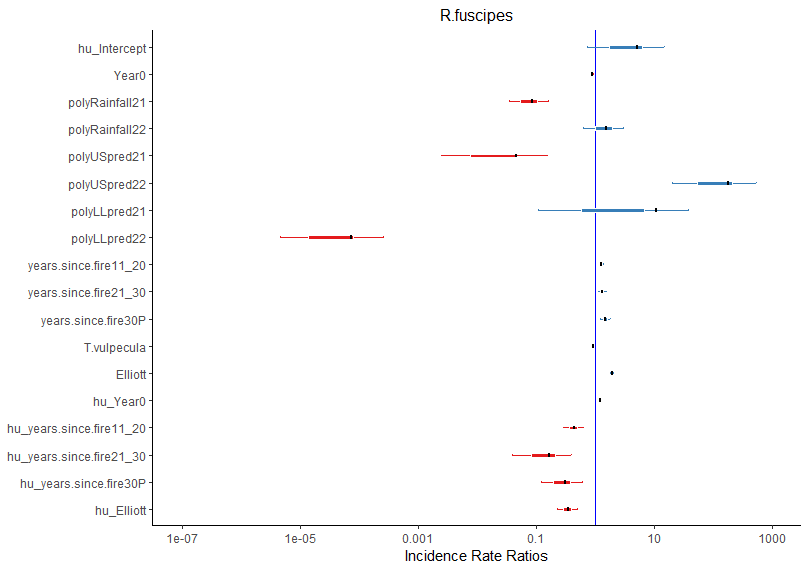

Supplement: S1 Fig — The blue vertical line represents the zero-effect line. Red horizontal lines represent negative effects, blue horizontal lines represent positive effects. The black dot represents the posterior mean. Lines that cross the zero-effect line represent non-significant results. (TIF) [file pone.0292919.s001.tif]

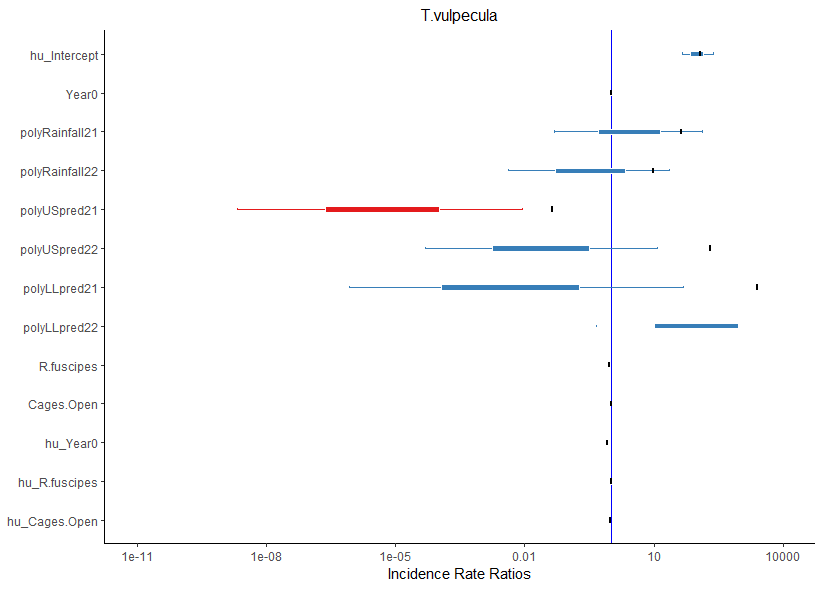

Supplement: S2 Fig — The blue vertical line represents the zero-effect line. Red horizontal lines represent negative effects, blue horizontal lines represent positive effects. The black dot represents the posterior mean. Lines that cross the zero-effect line represent non-significant results. (TIF) [file pone.0292919.s002.tif]

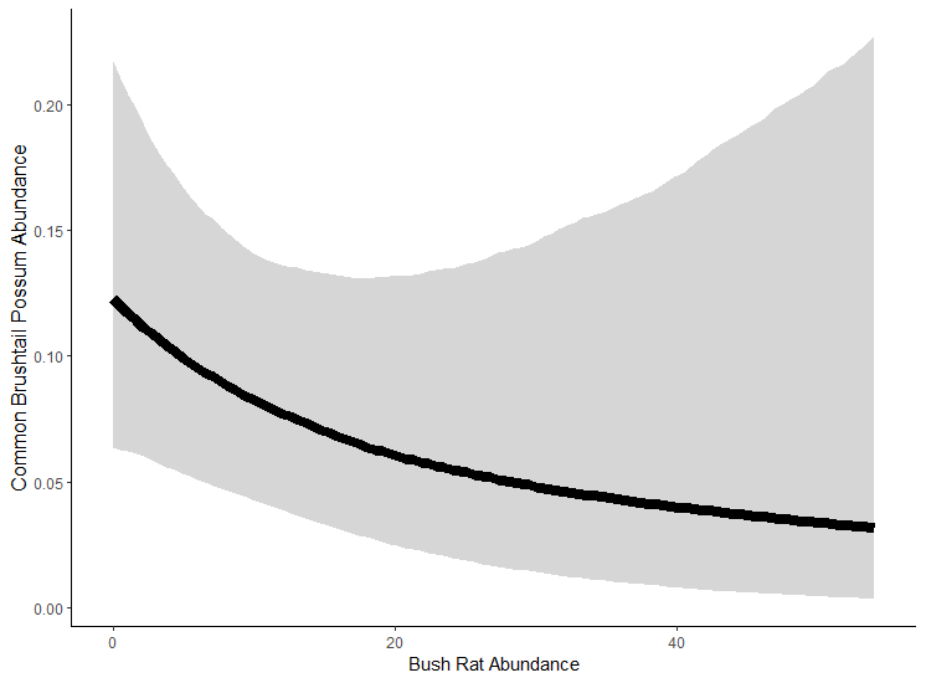

Supplement: S3 Fig — The shaded regions represent the 95% credible intervals. (TIF) [file pone.0292919.s003.tif]
