# Supplementary material for: Environmental variables influence patterns of mammal co-occurrence following introduced predator control
Source: PLoS One. 2023 Nov 30;18(11):e0292919. doi: 10.1371/journal.pone.0292919 (PMC10688647; doi:10.1371/journal.pone.0292919)
Supplement: Attachment — Submitted filename: kanishka_rebuttal_cooccurrence_plosone.docx [file pone.0292919.s004.docx]

Daniel de Paiva Silva, Ph.D.
Academic Editor
PLOS ONE

Dear Dr. de Paiva Silva,

Thank you for inviting us to submit a revised draft of our manuscript entitled, ‘Environmental variables influence patterns of mammal co-occurrence following introduced predator control’ to PLOS ONE.

We thank the editor and reviewers for taking the time to review our revised manuscript. In response to reviewer #3’s query, we have now made all of the data public on the Dryad Digital Repository ((DOI): doi:10.5061/dryad.gmsbcc2sh), including a text file with our R code for full transparency and reproducibility. We thank reviewer #3 for their comment.

We have included our full responses to the reviewers on the next page.

We believe that, with these revisions, our manuscript is now ready for publication in PLOS ONE.

Aurelie M. Kanishka
Fenner School of Environment and Society, Australian National University

On behalf of all authors

Reviewer #1: All points raised about review were answered coherently. Therefore, I believe in the potential of the manuscript.

Thank you for your support in our manuscript.

Reviewer #2: The authors will answer all the questions and doubts, to figure out now very best and most informative about the study area. I am grateful for the responses and parabenization of the authors for their work.

We are grateful that we have addressed all questions. Thank you for your support in our manuscript.

Reviewer #3: The authors addressed all my previous commentaries and significantly improved the clarity of their analytical approach, which was my main concern. This improvement, in turn, made their conclusions well-supported, and I congratulate the authors for their nice work. Before fully accepting the manuscript, my last remark is that their data still needs to be available on Dryad, as stated in the manuscript. Thus, I could not check if, together with the data, the author also made R scripts of their analytical approach available. If not, I encourage the authors to do so and fully contribute to reproducibility and transparency in science.

Thank you for your comments, and the advice given in the last round of revisions. We have now made our data public on the Dryad digital repository (link: https://doi.org/doi:10.5061/dryad.gmsbcc2sh.). Our software (i.e., R code) has also been made publicly available (link: https://doi.org/10.5281/zenodo.8378280).
